# The Prevalence of Irritable Bowel Syndrome Among Chinese University Students: A Systematic Review and Meta-Analysis

**DOI:** 10.3389/fpubh.2022.864721

**Published:** 2022-04-15

**Authors:** Weixin Yang, Xiao Yang, Xianghao Cai, Zhuoren Zhou, Huan Yao, Xingrong Song, Tianyun Zhao, Peng Xiong

**Affiliations:** ^1^Department of Public Health and Preventive Medicine, School of Medicine, Jinan University, Guangzhou, China; ^2^Department of Anesthesiology, Guangzhou Women and Children's Medical Center, Guangzhou Medical University, Guangzhou, China

**Keywords:** irritable bowel syndrome, prevalence, associated factors, Chinese university students, meta-analysis

## Abstract

**Background:**

Irritable bowel syndrome (IBS) has become a common public health issue among university students, impairing their physical and mental health. This meta-analysis aimed to examine the pooled prevalence of IBS and its associated factors among Chinese university students.

**Methods:**

Databases of PubMed, EMBASE, MEDLINE (*via* EBSCO), CINAHL (*via* EBSCO), Wan Fang, CNKI and Weipu (*via* VIP) were systematically searched from inception date to May 31, 2021. Meta-analysis was performed using random-effects models. Meta-regression and subgroup analysis were used to detect the potential source of heterogeneity.

**Key Results:**

A total of 22 cross-sectional studies (14 were in Chinese and 8 were in English) with 33,166 Chinese university students were included. The pooled prevalence of IBS was estimated as 11.89% (95% CI = 8.06%, 16.35%). The prevalence was 10.50% (95% CI = 6.80%, 15.87%) in Rome II criteria, 12.00% (95% CI = 8.23%, 17.17%) in Rome III criteria, and 3.66% (95% CI = 2.01%, 6.60%) in Rome IV criteria. The highest prevalence of IBS was 17.66% (95% CI = 7.37%, 36.64%) in North China, and the lowest was 3.18% (95% CI = 1.28%, 7.68%) in South China. Subgroup analyses indicated that gender, major, anxiety and depression symptoms, drinking and smoking behaviors were significantly associated with the prevalence of IBS. Meta-regression analyses suggested that region influenced prevalence estimates for IBS.

**Conclusions and Inferences:**

This meta-analysis illustrated that IBS is very common in Chinese university students. Regular screening, effective prevention, and appropriate treatments should be implemented to reduce the risk of IBS in this population. More future studies should be conducted in Northeastern and Southwestern parts of China.

## Introduction

Irritable bowel syndrome (IBS) is a chronic functional gastrointestinal disease characterized by altered bowel habits, abdominal discomfort or pain, and abdominal distension, without obvious structural or biochemical abnormalities (1) or organic etiology (2). A meta-analysis with 23 studies (*n* = 74,763) revealed that the prevalence of IBS was 6.5% in the general population in China (3). Though the pathophysiology is still unclear, IBS has been proved to be associated with multiple factors including abnormal gastrointestinal motility, visceral sensory abnormality, abnormal brain-gut regulation, inflammation, gastrointestinal infection, and stressful life events, etc. (4–6). Furthermore, recent evidences supported the negative psycho-influences to be the key role of the biopsychosocial model of IBS (4, 7–9). For instance, a meta-analysis showed the high rates of anxiety symptoms (39.1%) and disorders (23%), depression symptoms (28.8%) and disorders (23.3%) in IBS patients (10). Throughout the years, various criteria including the Manning criteria, the Rome I, Rome II, Rome III and Rome IV criteria, have been applied for diagnosis of IBS. Amongst them, the Rome III criteria (11) and Rome IV criteria (12) are the most commonly used currently.

University students are more likely to experience IBS–varied from 1.18% (13) to 33.3% (14) in China, might due to the psychological problems, unhealthy lifestyles, and a low level of health literacy (15). For instance, they are more prone to suffer from anxiety and depression symptoms, because of difficulties in terms of academic pressures, occupational choices, interpersonal conflict, and life goal decisions (16), which could cause gastrointestinal disorders through the brain-gut axis mechanism (17). A lack of physical exercise, irregular eating habits (i.e., not having breakfast), smoking, and drinking behaviors have also been found common in university students (18), which may contribute to the risk of IBS in this population. Moreover, due to various clinical examinations and constant medical treatment, IBS has been proved to be linked to physical problems like headache, chronic back or neck pain and diabetes (19), mental disorders like anxiety and depression (20) and obsessive-compulsiveness (21), sleep disorder (22), poorer academic achievements (23), lower quality of life (24, 25), social embarrassment due to diarrhea (a symptom of IBS) which restricts the patients being near a bathroom (26), and higher economic cost (24).

There has been a growing number of studies on IBS in Chinese university students, but the prevalence of IBS varied widely in existing studies. Precise epidemiological figures related to IBS prevalence are fundamental to inform preventive strategies in an evidence-based way. This study aimed to quantitatively evaluate the prevalence of IBS and its associated factors among Chinese university students *via* systematic review and meta-analysis.

## Materials and Methods

### Search Strategy and Selection Criteria

This study was performed according to the Preferred Reporting Items for Systematic Review and Meta-Analysis (PRISMA) Statement (27) and Meta-analyses Observational Studies in Epidemiology (MOOSE) guidelines (28). A systematic search was conducted in both English (PubMed, EMBASE, MEDLINE *via* EBSCO, CINAHL *via* EBSCO) and Chinese databases (Wan Fang, CNKI, Weipu *via* VIP) from their inception date to May 31, 2021. The searching terms were followed: (((Irritable OR spastic OR Mucous) AND (bowel OR colon OR colonic OR gastrointestinal)) OR IBS) AND (China OR Chinese OR mainland China OR Hong Kong OR Macau OR Macao OR Taiwan) AND (College OR University OR undergrad^*^). The search strategies in different databases were provided in Supplementary Table 1. The cited references of the identified publications were also searched manually to ascertain additional studies that may have been missed. The corresponding author would be contacted to get the essential information if needed.

**Figure 1:**
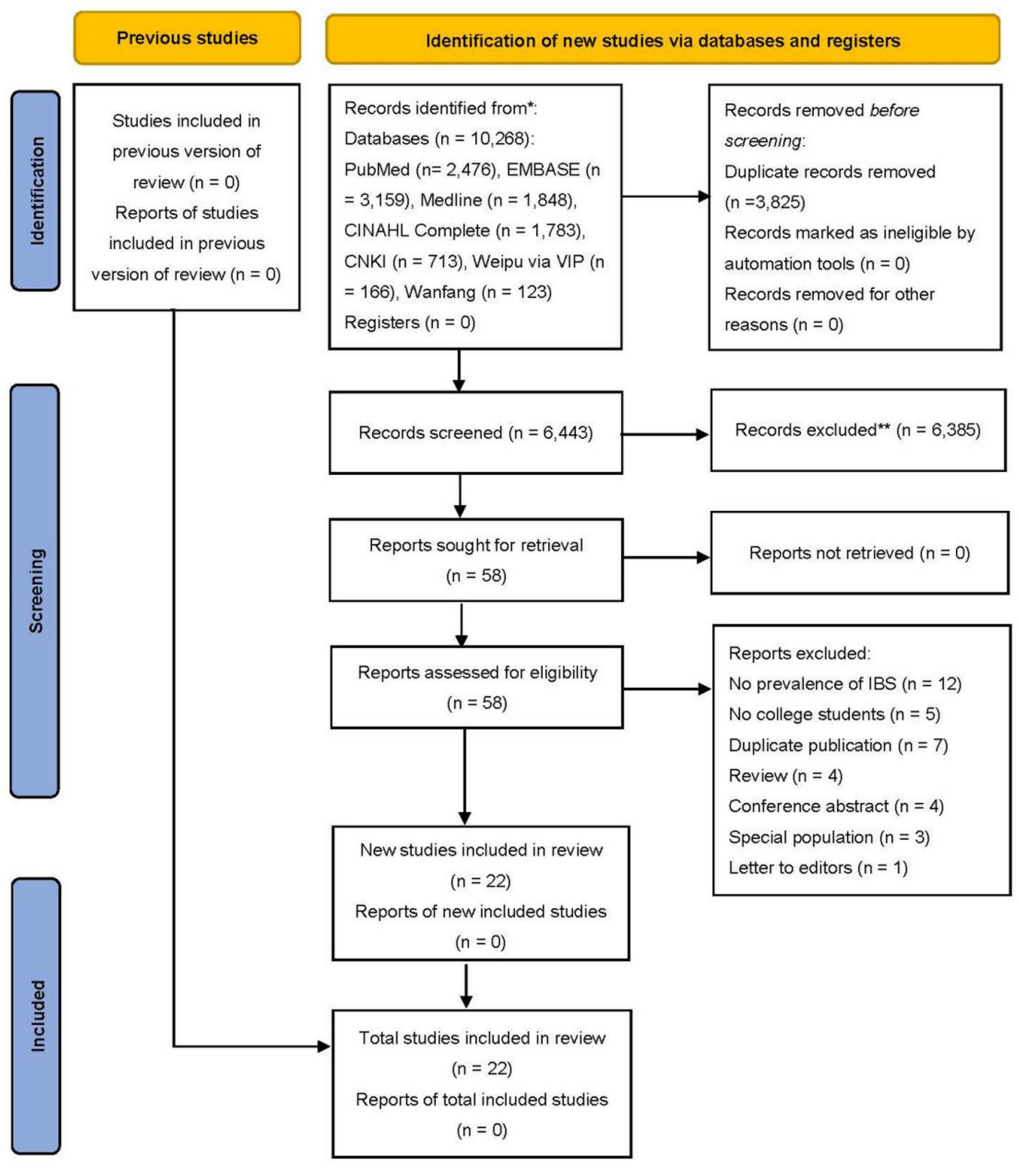
PRISMA flow chart of literature search and article selection process.

The titles and abstracts were initially screened and those that were obviously irrelevant were excluded. The full texts of the remaining articles were reviewed to find relevant studies that were finally included. The selection above was performed by two researchers (WX Yang and X Yang) independently and any discrepancies were resolved by discussing with the senior researcher (XH Cai). Figure 1 detailed the process of screening articles.

Studies were included if they met the following criteria: (1) original studies including cross-sectional and cohort studies; (2) participants should be full-time undergraduate students, junior college students, or postgraduates in China (including Hong Kong, Macao, and Taiwan); (3) reporting the prevalence of IBS with diagnostic criteria based on validated questionnaires or scales according to Manning or Rome criteria or International Classification of Diseases codes; (4) studies recruiting at least 50 subjects; (5) accessible full texts in English or Chinese. We excluded studies if they met the following criteria: (1) studies focused on special populations with medical conditions like gastritis or hepatitis; (2) studies without the prevalence of IBS reported; (3) full-texts not being available. If two or more papers were published based on the same dataset, only the one with the most complete information was included.

### Data Analysis

We used the “Checklist for Prevalence Studies” developed by the Joanna Briggs Institute for quality assessment (29, 30). The checklist consists of nine items, including (1) appropriate sampling frame, (2) appropriate sampling design, (3) adequate sample size, (4) detailed description of study subjects and setting, (5) sufficient coverage of sample, (6) valid methods for identifying the condition, (7) standard and reliable measurement of the condition (8) appropriate statistical analysis, and (9) adequate response rate. Each item was rated as either “yes”, “no”, “unclear”, or “not applicable”. Only the “yes” answer for each item receives a score of 1. Thus, final scores for each study could range from 0 to 9. Study quality was assessed by two researchers (WX Yang and X Yang) independently and any discrepancies were resolved by discussing with a third researcher (XH Cai).

Two researchers (WX Yang and X Yang) independently conducted data extraction, and any inconsistencies in the process were checked and resolved by involving a third investigator (XH Cai). The following information was extracted and tabulated: author, year of publication, sample characteristics {gender[Male/Female (M/F)], age [Mean ± Standard Deviation(SD)], grade, majors, educational level}, survey year, survey province, survey region, sampling method, total subjects, subjects with IBS, the prevalence of IBS, diagnostic criteria of IBS, subgroups, risk factors, and other significant results. Seven regions were identified in China, as shown in Supplementary Data 1. Majors were classified into “medicine” (clinical medicine, nursing, and health-related specialties set in the medical university), “non-medicine” or “mixed”. “Mixed” was defined as a mixture of different majors, which could not extract the specific major data in the paper. Only Rome III prevalence data was extracted if the study contained both Rome II and Rome III criteria.

The pooled prevalence of IBS was calculated as effect size (ES). Given the prevalence of IBS in most included studies (ranging from 0 to 20%) was close to the margins, the variance-stabilizing Freeman-Tukey double arcsine transformation was used to combine rates (31). Raw prevalence estimation was transformed and then multiple meta-analyses were performed with the transformed proportions using the random-effects model. These were then back-transformed to prevalence rates to facilitate interpretation of the outcomes and confidence interval (CI) (32). The *I*^2^ statistic was used to assess heterogeneity between the studies (low: *I*^2^ <25%, moderate: 25–50%, high: *I*^2^ > 50%) (33, 34). The funnel plot and Begg's test (35) were conducted to explore publication bias when there were at least 10 studies in the meta-analysis (36). The “*metaninf* ” command was used for sensitivity analysis *via* evaluating the effect of each study on overall estimates.

Subgroup analyses were conducted to examine the possible sources of heterogeneity according to the following categorical variables: (1) educational level: junior college vs. undergraduate vs. postgraduate; (2) gender: female vs. male; (3) majors: medicine vs. non-medicine vs. mixed; (4) regions: Central China vs. East China vs. North China vs. Northwest China vs. South China; (5) survey year: 2005–2010 vs. > 2010; (6) criteria: Rome II vs. Rome III vs. Rome IV; (7) anxiety: yes vs. no; (8) depression: yes vs. no; (9) drinking: yes vs. no; and (10) smoking: yes vs. no. To identify the factors associated with the prevalence of IBS in Chinese university students, pooled odd ratios (ORs) for potential influencing factors were calculated with a random-effects model.

Univariate meta-regression was performed to identify sources of between-study heterogeneity according to the following variables: educational level, gender ratio (M/F), major, region, survey year, criteria, anxiety proportion, depression proportion, drinking proportion, smoking proportion, and quality score. The significance level was set at *p* < 0.05 (two-tailed) for all analyses.

The Stata 14.0 (Stata Corporation, College Station, TX, USA) and Comprehensive Meta-Analysis Version 2.0 (Biostat, Inc., Englewood, New Jersey, USA) were administered to synthesize data.

## Results

### Study Characteristics

A total of 10,268 citations were initially searched in the databases, with 6,443 remaining after the removal of duplicates. After evaluating the title and/or abstract, 6,385 citations were removed for non-compliance with inclusion criteria. The full text of the remaining 58 citations was evaluated, and a total of 22 citations that met the criteria were included. Two citations with the same data were both included due to the different content in subgroup analyses and significant results, and the sample size of one citation was included when calculating the number of participants in this review. Finally, a total of 22 citations (14 in Chinese and 8 in English) with 33,166 Chinese university students were included in the analysis (Figure 1). All 22 studies reported the prevalence of IBS in university students, 20 reported risk factors for IBS, and 21 reported other significant results. A list of all included studies was presented in Supplementary Data 2. The characteristics of the study were summarized in Table 1.

**Table 1:** Characteristics of included studies.

**References**	**Survey year**	**Region, province**	**Sampling method**	**Quality score**	**Criteria of IBS**	**Gender (M/F)**	**Age (mean ±SD)**	**Major**	**Educational level and grade**	**Total subjects**	**Prevalence (%)**	**Other significant results**
Kong (37)	2006	East China, Shanghai	Random, stratified	8	Rome II	155/158	23.48 ± 2.46	Medicine	Undergraduate, postgraduate	313	13.42	• With the Rome II criteria, 8 cases were IBS-C (19.05%), 24 cases were IBS-D (57.14%), and 10 cases were IBS-M (23.81%). • Compared with the non-IBS group, scores of anxiety and depression were higher in the IBS group (*p* < 0.001).
Shen (38)	2006	Central China, Hubei	Stratified	9	Rome II	166/165	24.69 ± 2.10	Mixed	Undergraduate year 1, postgraduate year 1–2	331	15.4	• IBS was detected in 19.7% of the non-medical professional group, and 10.5% of the medical professional group, with significant differences seen between groups (*p* = 0.022). • Compared with the non-IBS group, scores of anxiety and depression were higher in the IBS group.
Dai (39)	2007	East China, Zhejiang	Cluster	9	Rome II, Rome III	517/604	21.8 ± 3.2	Mixed	Undergraduate	1,121	4.7 (Rome II), 10.4 (Rome III)	• With the Rome II criteria, 8 cases were c-IBS (14.81%), 12 cases were d-IBS (22.22%), and 34 cases were a-IBS (62.96%). With the Rome III criteria, 18 cases were IBS-C (14.63%), 30 cases were IBS-D (24.39%), 59 cases were IBS-M (47.97%), and 16 cases were IBS-U (13.01%).
Shen (5)	2006	Central China, Hubei	Stratified	9	Rome II	241/250	24.13 ± 2.069	Mixed	Undergraduate year 1	491	15.7	• IBS was associated with anxiety (*p* < 0.001) and depression (*p* < 0.001).
Dong (40)	2009	East China, Shandong	Random	9	Rome II	917/1209	20.64 ± 1.593	Mixed	Undergraduate	2,126	7.85	• The IBS group scored higher in anxiety (*p* < 0.001), depression (*p* < 0.001) and lower in exercise frequency (*p* = 0.007) compared to the non-IBS group. • With the Rome III criteria, 61 cases were IBS-C (36.5%), 51 cases were IBS-D (31.1%), 40 cases were IBS-M (23.9%), and 25 were non-IBS cases (8.5%).
Liu (41)	2009	East China, Jiangxi	Cluster	8	Rome III	392/568	19.68 ± 2.14	Mixed	Junior college year 1–2	960	12.81	• With the Rome III criteria, 58 cases were IBS-C (47.51%), 23 cases were IBS-D (18.34%), and 42 cases were IBS-M (34.25%). • IBS was associated with health-related majors (*p* < 0.01) and higher grade (*p* < 0.01). • Compared with the non-IBS group, the IBS group has a higher prevalence of anxiety and depression.
Shi (42)	2008	Central China, Henan	Cluster	8	Rome III	414/1,520	19.7 ± 1.4	Medicine	Undergraduate	1,934	32.1	• With the Rome III criteria, 203 cases were IBS-C (32.69%), 168 cases were IBS-D (27.05%), and 250 cases were IBS-M (40.26%). • Higher height (*p* = 0.018), shorter sleep time (*p* = 0.024) and weight loss (*p* < 0.001) were related to IBS in females.
Jiang (13)	/	South China, Guangdong	Stratified	7	Rome III	161/178	20.12 ± 0.63	Mixed	Junior college, undergraduate	339	1.18	• Mental factors were related to functional gastroenteropathy (*p* < 0.05).
Lin (43)	/	North China, Hebei	Stratified	8	Rome III	388/1,370	18–24 (age range)	Medicine	Junior college, undergraduate	1,758	8.99	• IBS was associated with female (*p* = 0.049), educational level (*p* < 0.001), major (*p* = 0.026).
Wu (44)	2011	Central China, Hubei	Stratified	6	Rome III	86/137	20.26	Medicine	Undergraduate	223	6.7	• IBS was associated with lack of physical exercise (*p* = 0.035), spicy diet (*p* = 0.009), anxiety (*p* = 0.049), gastrointestinal infection (*p* = 0.002), antibiotics taking (*p* = 0.046), painkillers taking (*p* = 0.009), lack of amusement (*p* = 0.017) and parents having the same symptoms (*p* = 0.012).
Dong (45)	2012	East China, Shandong	Random	9	Rome III	2,215/2,423	20.768 ± 1.509	Mixed	Undergraduate	4,638	8.34	• IBS was associated with anxiety (*p* = 0.002) and depression (*p* = 0.045). • With the Rome III criteria, 150 cases were IBS-C (38.76%), 189 cases were IBS-D (48.84%), and 48 cases were IBS-M (12.40%).
Li (46)	2010–2011	East China, Zhejiang	/	8	Rome III	967/903	21.34 ± 2.56	Mixed	Undergraduate year 1–4, postgraduate year 1	1,870	6.9	/
Liu (14)	2014	North China, Beijing	Stratified	8	Rome III	196/571	23.26 ± 2.88	Medicine	Undergraduate and postgraduate, year 1–7	767	33.3	• For females, the IBS participants scored higher in anxiety (*p* = 0.015). • The IBS group scored higher in emotional neglect than the non-IBS group (*p* = 0.045). • Medical students with IBS scored higher on the PSQI than those without IBS (*p* < 0.001 in females, *p* = 0.014 in males). • With the Rome III criteria, 15 cases were IBS-C (5.88%), 79 cases were IBS-D (30.98%), 112 cases were IBS-M (43.92%), and 49 cases were IBS-U (19.22%).
Yang (47)	2014	North China, Beijing	Stratified, cluster	7	Rome III	196/571	23.26 ± 2.88	Medicine	Undergraduate and postgraduate, year 1–7	767	33.3	• With the Rome III criteria, 15 cases were IBS-C (5.88%), 79 cases were IBS-D (30.98%), 112 cases were IBS-M (43.92%), and 49 cases were IBS-U (19.22%). • Compared with the non-IBS group, the score of the life stress questionnaire was higher in the IBS group (*p* < 0.05).
Zhang (48)	2012–2013	Northwest China, Xinjiang	Stratified	9	Rome III	248/193	24.57 ± 2.02	Mixed	Postgraduate year 1-3	441	11.56	• IBS prevalence was higher in groups of females (*p* = 0.021), eating cold food frequency ≥ 3 times a week (*p* < 0.001), eating dairy product frequency ≥3 times a week (*p* = 0.001), eating high-fiber foods frequency <4 times a week (*p* = 0.011), physical activity time <4 h a week (*p* = 0.029), insomnia frequency ≥ 3 times a week (*p* < 0.001), anxiety (*p* = 0.013) and depression (*p* = 0.002).
Li (49)	2015	North China, Beijing	Stratified	8	Rome III	425/282	20.28 ± 1.46	Mixed	Undergraduate year 1–4	707	16.7	• With the Rome III criteria, 16 cases were IBS-C (13.6%), 40 cases were IBS-D (33.9%), 54 cases were IBS-M (45.8%), and 8 cases were IBS-U (6.8%). • IBS was detected differently in females (20.2%) and males (14.4%, *p* = 0.041). • Compared to the healthy control group, participants in the IBS group reported higher scores of somatization symptom (*p* < 0.001), test anxiety (*p* = 0.026), negative life events (*p* = 0.002), and lower scores of physical symptoms and organ function (*p* < 0.001), psychological symptoms and negative emotions (*p* = 0.036), role activities and social adaptation (*p* = 0.008), social resources and social contact (*p* = 0.027) of SRHMS. • Gender, smoking, eating chilies, high physical-sensitive independently related to IBS.
Wang (50)	2013	Northwest China, Inner Mongolia	Stratified	7	Rome III	1,667/4,438	21 ± 1.5	Mixed mixed	Undergraduate year 1–3	6,105	29.5	• With the Rome III criteria, 364 cases were IBS-C (20.22%), 866 cases were IBS-D (48.11%), 322 cases were IBS-M (17.89%), and 248 cases were IBS-U (13.78%). • IBS was detected differently in females (31.3%) and males (24.8%, *p* < 0.001). • IBS was associated with lose weight (*p* < 0.001), anxiety (*p* < 0.001) and depression (*p* = 0.026).
Yang (51)	2014–2015	South China, Guangdong	Stratified	7	Rome III	/	/	Mixed	Undergraduate year 1–3	2,847	7.38	• With the Rome III criteria, 76 cases were IBS-C (36.19%), 101 cases were IBS-D (48.10%), and 33 cases were IBS-M (15.71%).
Chen (52)	2016	East China, Shanghai	Stratified, cluster	7	Rome III	0/468	19.60 ± 1.20	Nursing	Junior college year 1–3	468	17.31	• With the Rome III criteria, 43 cases were IBS-C (53.09%), 19 cases were IBS-D (23.46%), 14 cases were IBS-M (17.28%), and 5 cases were IBS-U (6.17%). • IBS was associated with spicy diet (*p* = 0.014), sleep disorder (*p* = 0.047) and lower grade (*p* = 0.008).
Liu (53)	2019	South China, Guangxi	Cluster	8	Rome IV	593/2,033	19.22 ± 1.03	Health related	Junior college year 1	2,626	2.7	• IBS was associated with alcohol consumption (*p* = 0.021), dairy intake (*p* = 0.001), fatigue (*p* = 0.003), poor mood situation (*p* < 0.001) in healthy freshmen.
Chen (54)	2016	East China, Taiwan	Convenience	7	Rome III	0/1,894	21.59 ± 1.40	Medicine, non-medicine	Undergraduate year 2–4	1,894	10.1	• Compared with the non-IBS female students, IBS female students had higher levels of stress and lower QoL. • IBS in females was associated with dysmenorrhea (*p* < 0.001), food avoidance (*p* < 0.001), class absenteeism (*p* < 0.001), and the lower physical domain of QoL (*p* < 0.001).
Zhang (55)	2018–2019	East China, Jiangsu	Cluster	8	Rome IV	533/674	/	Medicine, non-medicine	Undergraduate year 1–4	1,207	5.1	• IBS was associated with irregular menstruation (*p* < 0.05) and previous history of gastroenteritis (*p* < 0.05).

*Gender (M/F), Gender (Male/Female); Junior college students, students studying in 3-year college degree: Undergraduate students, students studying in 4-year or 5-year bachelor degree; Postgraduate students, students studying in master degree; Under Rome II criteria, IBS-C, irritable bowel syndrome with constipation; IBS-D, irritable bowel syndrome with diarrhea; IBS-M, irritable bowel syndrome mixed; IBS-U, irritable bowel syndrome un-subtyped; Under Rome III criteria: c-IBS, constipation predominant irritable bowel syndrome; d-IBS, diarrhea predominant irritable bowel syndrome; a-IBS, alternative irritable bowel syndrome; SRHMS, self-rated health measurement scale; PSQI, Pittsburgh sleep quality index; QoL, The World Health Organization Quality of Life-BREF Questionnaire*.

### Quality Assessment and Publication Bias

The scores of study quality assessment ranged from 6 to 9 with a mean score of 7.8. The most common missing items in the studies included the reports of detailed information about the study subjects and the detailed descriptions of the process of collecting data or the professionalism of the person collecting the data (Supplementary Table 2).

No significant publication bias was found by the funnel plot (Supplementary Figure 1) in the 21 studies. Begg's test (*z* = 1.48, *p* = 0.139) also did not detect significant bias.

### Prevalence of IBS

**Figure 2:**
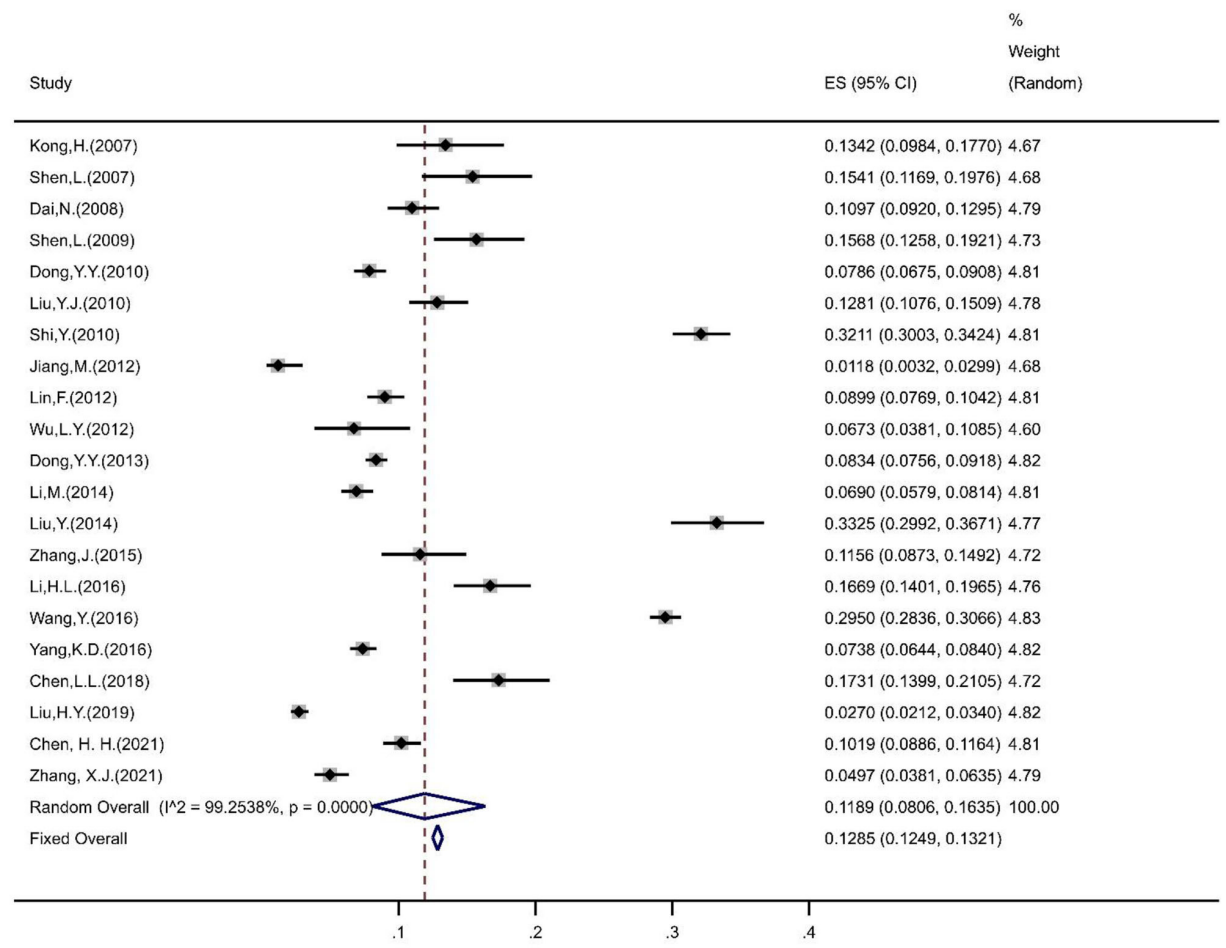
Forest plot of prevalence of IBS in Chinses university students. ES, effect size.

The pooled prevalence of IBS in Chinese university students was estimated to be 11.89% (95% CI = 8.06%, 16.35%) based on the random-effects model (Figure 2). The sensitivity analysis indicated that no study affected the prevalence estimate by more than 1%, suggesting that the overall prevalence estimate was powered to the methodological quality of each research study included.

Among them, the prevalence of IBS was 8.18% (95% CI = 3.66%, 17.26%) for junior college students, 12.14% (95% CI = 8.02%, 17.96%) for undergraduate students, and 12.74% (95% CI = 10.10%, 15.94%) for postgraduate students. The prevalence of IBS was 13.14% (95% CI = 9.22%, 18.39%) in females and 10.17% (95% CI = 6.39%, 15.80%) in males. The prevalence of IBS in medical, non-medical, and mixed majors were 11.91% (95% CI = 8.13%, 17.12%), 11.35% (95% CI = 8.90%, 14.37%), and 6.48% (95% CI = 2.13%, 18.05%), respectively. In terms of regions, the prevalence of IBS among university students was 15.93% (95% CI = 8.28%, 28.47%) in Central China, 10.50% (95% CI = 8.40%, 13.06%) in East China, 17.66% (95% CI = 7.37%, 36.64%) in North China, 19.10% (95% CI = 7.02%, 42.46%) in Northwest China, and 3.18% (95% CI = 1.28%, 7.68%) in South China. Under different diagnostic criteria, the prevalence of IBS was 10.50% (95% CI = 6.80%, 15.87%) in Rome II, 12.00% (95% CI = 8.23%, 17.17%) in Rome III, and 3.66% (95% CI = 2.01%, 6.60%) in Rome IV.

The prevalence of IBS in people with anxiety and depression symptoms was 17.31% (95% CI = 8.36%, 32.44%) and 17.34% (95% CI = 8.32%, 32.68%), respectively. The prevalence of IBS was 11.11% (95% CI = 6.40%, 18.60%) in people who drank, 18.10% (95% CI = 5.59%, 45.18%) in people who smoke.

### Associated Factors With IBS

Subgroup analysis found that a higher prevalence of IBS was significantly associated with postgraduate students, females, medical majors, anxiety, and depression symptoms, drinking and smoking behaviors (all *P* < 0.001) (Table 2, Supplementary Table 3). The survey region, survey year, diagnostic criteria were also significantly associated with the prevalence of IBS (all *P* < 0.001) (Table 2, Supplementary Table 3).

**Table 2:** Subgroup analyses of the pooled prevalence of IBS.

**Subgroup analysis**	**Studies (*n*)**	**Sample size (*n*)**	**Pooled prevalence (%)**	**95% CI**		***I*^2^(%) within subgroup**	***p-*value across subgroups**
				**Lower**	**Upper**		
Educational level							**<0.001**
Junior college	4	5,063	8.18	3.66	17.26	98.24	
Undergraduate	13	24,202	12.14	8.02	17.96	99.27	
Postgraduate	3	925	12.74	10.10	15.94	40.36	
Gender							**<0.001**
Female	16	18,252	13.14	9.22	18.39	98.72	
Male	14	9,279	10.17	6.39	15.80	98.02	
Major							**<0.001**
Medicine	15	10,046	11.91	8.13	17.12	98.02	
Non-medicine	10	11,077	11.35	8.90	14.37	93.49	
Mixed	5	12,061	6.48	2.13	18.05	99.55	
Region							**<0.001**
Central China	4	2,979	15.93	8.28	28.47	97.39	
East China	7	14,597	10.50	8.40	13.06	92.56	
North China	3	3,232	17.66	7.37	36.64	99.03	
Northwest China	2	6,546	19.10	7.02	42.46	98.30	
South China	3	5,812	3.18	1.28	7.68	97.00	
Survey year							**<0.001**
2005–2010	7	7,276	14.42	8.48	23.46	98.65	
2010–2021	12	23,793	10.60	6.42	17.00	99.38	
Criteria							**<0.001**
Rome II	5	4,382	10.50	6.80	15.87	94.58	
Rome III	15	26,072	12.00	8.23	17.17	99.21	
Rome IV	2	3,833	3.66	2.01	6.60	92.00	
Anxiety							**<0.001**
No	6	11,058	8.61	3.72	18.66	99.31	
Yes	6	2,640	17.31	8.36	32.44	98.02	
Depression							**<0.001**
No	5	10,743	9.11	3.60	21.19	99.43	
Yes	5	2,798	17.34	8.32	32.68	98.10	
Drinking							**<0.001**
No	4	3,570	10.78	3.51	28.62	99.05	
Yes	4	970	11.11	6.40	18.60	85.58	
Smoking							**<0.001**
No	4	2,815	14.70	6.09	31.43	98.65	
Yes	4	307	18.10	5.59	45.18	87.47	

*Boldface indicates statistical significance (p < 0.05).*

*CI, confidence interval*.

In the univariate meta-regression, region (*p* = 0.01) was identified as a significant moderator that contributed to heterogeneity between the studies. However, educational level (*p* = 0.66), gender ratio (*p* = 0.19), major (*p* = 0.05), survey year (*p* = 0.57) criteria (*p* = 0.41), anxiety proportion (*p* = 0.29), depression proportion (*p* = 0.14), drinking proportion (*p* = 0.64), smoking proportion (*p* = 0.71) and quality score (*p* = 0.48) were non-significant moderators (Table 3).

**Table 3:** Univariate meta-regression analyses of prevalence of IBS.

	**Variable**	**Coefficient**	**SE**	***T***	***P* > |t|**	**95%CI**	
					**Lower**	**Upper**
Univariate analysis	Educational level	0.02	0.04	0.45	0.66	−0.07	0.11
	Gender ratio (M/F)	−0.09	0.06	−1.36	0.19	−0.23	0.05
	Major	−0.09	0.04	−2.06	0.05	−0.18	0.00
	Region	0.03	0.01	2.84	**0.01**	0.01	0.06
	Survey year	−0.03	0.05	−0.58	0.57	−0.12	0.07
	Criteria	−0.03	0.04	−0.84	0.41	−0.01	0.05
	Anxiety proportion	−0.21	0.17	−1.23	0.29	−0.68	0.26
	Depression proportion	−0.35	0.18	−1.96	0.14	−0.92	0.22
	Drinking proportion	−0.16	0.32	−0.51	0.64	−1.17	0.84
	Smoking proportion	−0.15	0.33	−0.43	0.71	−1.59	1.30
	Quality score	0.02	0.02	0.72	0.48	−0.03	0.06

*Boldface indicates statistical significance (p < 0.05). CI, confidence interval; M/F, male/female*.

Items of sleep disorders (OR = 1.48, 95% CI = 1.02, 2.15), anxiety (OR = 2.35, 95% CI = 2.03, 2.72), depression (OR = 2.15, 95% CI = 1.88, 2.47), and gender (OR = 1.36, 95% CI = 1.08, 1.69) were statistically associated with the development of IBS in Chinese university students (Supplementary Table 4), which was also found in the corresponding subgroup analyses.

## Discussion

To the best of our knowledge, this is the first systematic review and meta-analysis to estimate the pooled prevalence of IBS among Chinese university students, including 22 studies with 33,166 subjects. The major findings are: (1) the pooled prevalence of IBS among Chinese university students was 11.89% (95% CI = 8.06%, 16.35%); (2) the prevalence of IBS was significantly associated with educational level, gender, major, region, survey year, diagnostic criteria, anxiety, depression, drinking, smoking, and sleep disorders.

The prevalence of IBS in our study was approximate to that of 10.9% in American university students (56) and 10.7% in Japanese university students (57), but higher than that in Korean college students (5.7%) (58), lower than that in Pakistan college students (34%) (59). The possible explanations might be the difference in culture, diet habits, physical characteristics, academic and socioeconomic stress across countries.

Students with psychological disorders such as anxiety and depression had an increased likelihood of IBS comorbidity compared to those without. The link between psychosocial factors and gastrointestinal function (motility, sensation, inflammation) could be explained by the brain-gut axis (17). Specifically, this implies a bidirectional connection system between the gastrointestinal tract and the brain, through neural, neuroimmune and neuroendocrine pathways. In this model, individuals with increased central nervous system (CNS) arousal such as those with anxiety and depression, could experience gastrointestinal distress and increased gastrointestinal motility *via* CNS-mediated sympathetic outflow (60), leading to the destruction in the intestinal mucosal barrier (61) and the change of transport in the small intestine and even the entire gastrointestinal tract (62) and, resulting in gastrointestinal symptoms (cramping and pain, etc.) of IBS.

Our study found that the prevalence of IBS was higher among female students. This discrepancy could be attributed to several factors. First, the difference in the secretion of sex hormones contributes to the gender difference in the modulation of IBS. For example, androgens, higher in males, possibly could reduce visceral pain through enhancing TRPM8 expression and/or activity (63). TRPM8 is suggested to possess anti-nociceptive roles in the intestine (64) and ligands of TRPM8 such as peppermint are believed to possess analgesic effects in IBS patients (65, 66). As for females, the higher level of hormones like estrogen contributes to the development of IBS. It is reported that estrogens inhibit colonic smooth muscle contraction *via* a non-genomic mechanism involving cell membrane coupling (67), leading to the higher occurrence of IBS-related symptoms including abdominal distension, bloating, infrequent stools and hard stools (68). Estrogens promote activation of mast cells (68), which are found to be associated with IBS through increasing intestinal nerve sensitization (69). Second, for females, increased prostaglandins during the menstrual cycle could induce diarrhea syndrome, one of the IBS symptoms, through enhanced intestinal secretion and altered electrolyte absorption (70). Third, women are more vulnerable to experience life stress, anxiety, and depression symptoms (71, 72), which are associated with a higher incidence of IBS.

In terms of majors, a higher prevalence of IBS was estimated in the medical students. This might be due to the long length of schooling, high load from the academy and clinical practice, high level of psychological stress exposure like severe anxiety and depression (73) and sleep disturbances (74). It was proved that the stress was associated with the development of IBS through stimulating the hypothalamic-pituitary-adrenal (HPA) axis and triggering the release of some substances including corticotrophin-releasing factor (CRF), adreno-cortico-tropic-hormone (ACTH), and cortisol, which affect gut function through the composition and the growth of microbiota, and stimulate the sympathetic nervous system (SNS) (75). Sleep disorder, such as insomnia, was associated with a 24-h increase of ACTH and cortisol secretion (76). Furthermore, the symptoms of IBS, such as abdominal pain, might activate the SNS and then reduce sleep efficiency (76, 77).

Our results revealed that the prevalence of IBS in university students was higher in Northwest China (19.10%, 95% CI: 7.02–42.46%), North China (17.66%, 95% CI: 7.37–36.64%) and Central China (15.93%, 95% CI: 8.28–28.47%), followed by East China (10.50%, 95% CI: 8.40–13.06%) and South China (3.18%, 95% CI: 1.28–7.68%). This might be due to the varied territory, climate, diet, traditional customs, the development of socioeconomic and employment prospects across China (48). In the coastal regions–East China and South China, the relatively more moderate climate might benefit people's health and the superior socioeconomic conditions effectively alleviate the psychological stress for university students there (78), leading to a lower prevalence of IBS.

Be consistent with the results of previous studies (79, 80), the prevalence estimation in the Rome III criteria group (12.00%) was higher than groups of Rome II (10.50%) and Rome IV criteria (3.66%). Diagnosis of IBS can be challenging. Compared to Rome III, Rome II criteria examine a 12-week period duration in the past 12 months, less than a continuous 6-month period, thus expanding the scope of diagnosis and being more stringent (81). Rome IV criteria requires that abdominal pain occurs on average at least 1 day per week while only 3 days a month were required in Rome III criteria. This might be the most important factor accounting for a reduction in the estimated prevalence of IBS from Rome III to Rome IV (82). Dai et al. (39) suggested that the choice between Rome II and Rome III criteria may affect the IBS diagnosis in females more than males. Another diagnosis of IBS–Manning criteria was regarded to be applied to the private housing group rather than the public housing group (83). Studies showed that Manning criteria was more appropriate for females (84), but less sensitive for males (85, 86). The applicable diagnostic criteria seem different according to the research population.

Furthermore, university students with drinking and smoking behaviors were more likely to report IBS. It was explained that alcohol could decrease muscle movements, which helps retain the food for further digestion in the small intestine and reduce the frequency and strength of muscle contractions in a segment of the rectum. This could further reduce the transit time and the compaction of the intestinal content. In addition, alcohol interferes with the activity of lactase, which breaks down the milk sugar lactose, resulting in lactose intolerance. Thus, diarrhea was frequently observed in alcoholics. Alcohol also inhibits some enzymes that participate in the metabolism of foreign organic substances in the gut. It directly disturbs the integrity of the mucosal epithelium and induces the release of noxious signaling molecules, which could damage the small blood vessels of capillaries in the intestinal mucosa and induce blood clotting. The resulting lesions allow large molecules, such as endotoxins and other bacterial toxins, to enter the bloodstream and the lymph. Therefore, alcohol-induced digestive disorders and mucosal damage in the gastrointestinal (GI) tract cause the change in the frequency and appearance of the stool, abdominal pain and bloating (87), which were the symptoms of IBS. For smokers, nicotine stimulates the sympathetic nerve to inhibit the movement of the disinfectant tract and the secretion of the gland, resulting in gastrointestinal emptying delay and absorption dysfunction. Another explanation could be that oxygen-free radicals from smoking could enhance lipid peroxidation, implicated with gastrointestinal dysfunction (88).

The results of this meta-analysis have implications for future research. The prevalence of IBS among university students in certain regions such as Northeast and Southwest China, needs further study for the overall estimation with greater precision. More population-based studies using Rome IV criteria are required to explore the appropriateness of this criteria on Chinese university students. Future longitudinal studies are needed to be adopted to establish the causal relationships between IBS and potential influencing factors, which are greatly warranted for intervention development.

The findings of this meta-analysis should be interpreted with caution due to several limitations. First, the 22 included studies involved only 14 of 34 provincial-level administrative regions in China, which limited the generalizability of the findings to all university students in China. Second, although subgroup analyses somewhat mitigated this limitation (89), heterogeneity was impossible to avoid in the meta-analysis of epidemiological studies. Third, the potential association between IBS and some factors, such as frequency of exercise, could not be examined in the subgroup analyses due to incomplete data or inconsistent reporting forms in most included studies. The miss of studies only exploring the associated factors without the prevalence of IBS might lead to the insufficiency of data on the analyses of associated factors of IBS. Therefore, our results of associated factors of IBS needed to be treated with caution. Finally, the causal inference between IBS and other factors was not allowed because of the cross-sectional design in all included studies.

## Conclusions

This systematic review and meta-analysis showed that IBS was common (11.89%) in Chinese university students. The prevalence varied considerably in some instances, according to educational levels, geographic region, criteria used to define IBS. There are many associated factors of IBS, including female gender, majoring in medicine, anxiety, depression, drinking and smoking behavior. Further research should build on our findings and develop effective strategies for preventing and treating IBS in this population.

## Data Availability Statement

The original contributions presented in the study are included in the article/Supplementary Material, further inquiries can be directed to the corresponding authors.

## Author Contributions

PX: conceptualization, methodology, software, resources, writing-original draft, writing-review and editing, supervision, project administration, and funding acquisition. TZ: conceptualization, methodology, and funding acquisition. WY: methodology, validation, formal analysis, investigation, data curation, and writing–original. XY: validation, formal analysis, investigation, data curation, writing-original draft, and visualization. XC: methodology, investigation, draft, and visualization. ZZ and HY: methodology, investigation, and visualization. XS: review and revision. All authors contributed to the article and approved the submitted version.

## Funding

PX was supported by grants Moral Education Research Project for Teaching Science of Education Department of Guangdong Province (NO. 2019JKDY005) and National Natural Science Foundation of China (NO. 31970990). TZ was supported by grants Guangzhou Institute of Pediatrics/Guangzhou Women and Children's Medical Center funds (NO. GCP-2018-001) and Program of Guangzhou Municipal Science and Technology Bureau (NO. 201803010025). The funding body had no role in the study design, data collection, data analysis, data interpretation, the writing of the manuscript and the decision to submit the paper for publication. The research presented in this paper is that of the authors and does not reflect the official policy of Department of Public Health and Preventive Medicine, School of Medicine, Jinan University, Guangzhou, China. PX has full access to all the data in the study and takes responsibility for the integrity of the data and the accuracy of the data analysis.

## Conflict of Interest

The authors declare that the research was conducted in the absence of any commercial or financial relationships that could be construed as a potential conflict of interest.

## Publisher's Note

All claims expressed in this article are solely those of the authors and do not necessarily represent those of their affiliated organizations, or those of the publisher, the editors and the reviewers. Any product that may be evaluated in this article, or claim that may be made by its manufacturer, is not guaranteed or endorsed by the publisher.
