# Supplementary material for: The Prevalence of Irritable Bowel Syndrome Among Chinese University Students: A Systematic Review and Meta-Analysis
Source: Front Public Health. 2022 Apr 15;10:864721. doi: 10.3389/fpubh.2022.864721 (PMC9051230; doi:10.3389/fpubh.2022.864721)
Supplement: Supplementary file 1 [file Data_Sheet_1.docx]

Supplementary Material

# Supplementary Data

**Supplementary Data 1.** Regional division of Chinese geography

North China: Beijing municipality, Tianjin municipality, Hebei province, Shanxi province, central Inner Mongolia (Hohhot city, Wulanchabu city, Baotou city)

Northeast China: Heilongjiang province, Jilin province, Liaoning province, eastern Inner Mongolia (Xilin Gol League, Chifeng city, Tongliao city, Xingan League, Hulun Buir city);

East China: Shanghai municipality, Jiangsu province, Zhejiang province, Anhui province, Jiangxi province, Shandong province, Fujian province, and Taiwan province;

Central China: Henan province, Hubei province, Hunan province;

South China: Guangdong province, Guangxi Zhuang Autonomous Region, Hainan province, Hong Kong Special Administrative Region, Macao Special Administrative Region;

Southwest China: Chongqing municipality, Sichuan province, Guizhou province, Yunnan province, Tibet Autonomous Region;

Northwest China: Shaanxi province, Gansu province, Qinghai province, Ningxia Hui Autonomous Region, Xinjiang Uygur Autonomous Region, western Inner Mongolia Autonomous Region (Alxa League, Bayannur city, Wuhai city, Ordos city)

**Supplementary Data 2.** List of all included studies

1.Kong H, Shen L, Hou XH. (2007) Prevalence of irritable bowel syndrome among medical students in different grades: the effect of anxiety and depression [In Chinese]. *J Clin Intern Med*. *24*(12): 825-27 <https://doi.org/10.3969/j.issn.1001-9057.2007.12.011>.

2.Shen L, Kong H, Hou XH. (2007) An Epidemiologic Study of Irritable Bowel Syndrome in Graduates of Different Specialties [In Chinese]. *Chin J Gastroenterol*. *12*(01): 14-18 <https://doi.org/10.3969/j.issn.1008-7125.2007.01.004>.

3.Dai N, Cong Y, Yuan H. (2008) Prevalence of irritable bowel syndrome among undergraduates in Southeast China. *Dig Liver Dis*. *40*(6): 418-24 <https://doi.org/10.1016/j.dld.2008.01.019>.

4.Shen L, Kong H, Hou X. (2009) Prevalence of irritable bowel syndrome and its relationship with psychological stress status in Chinese university students. *J Gastroenterol Hepatol*. *24*(12): 1885-90 <https://doi.org/10.1111/j.1440-1746.2009.05943.x>.

5.Dong YY, Zuo XL, Li CQ, Yu YB, Zhao QJ, Li YQ. (2010) Prevalence of irritable bowel syndrome in Chinese college and university students assessed using Rome III criteria. *World J Gastroenterol*. *16*(33): 4221-26 <https://doi.org/10.3748/wjg.v16.i33.4221>.

6.Liu YJ, Xiong LS, Cui MC. (2010) The prevalence and influencing factors of irritable bowel syndrome among students in a vocational college in Jiangxi Province [In Chinese]. *Chin J Sch Health*. *31*(11): 1393-94 <https://doi.org/10.16835/j.cnki.1000-9817.2010.11.049>.

7.Shi Y, Liu LM, Zhang ZX. (2010) Current situation of irritable bowel syndrome in nursing and clinical medicine undergraduates [In Chinese]. *Zhengzhou Da Xue Xue Bao Yi Xue Ban*. *45*(05): 829-31 <https://doi.org/10.13705/j.issn.1671-6825.2010.05.025>.

8.Jiang M, Wang L, Liu X, Hu HX, Liang ZX, Zhang JA. (2012) The distribution and influencing factors offunctional gastrointestinal diseases in college students [In Chinese]. *Chin J Clinicians (Electronic Edition)*. *6*(17): 5281-82 [www.cnki.com.cn/Article/CJFDTotal-ZLYD201217082.htm](http://www.cnki.com.cn/Article/CJFDTotal-ZLYD201217082.htm).

9.Lin F, Li ZT. (2012) Investigation of functional gastrointestinal diseases among students of Tangshan Medical College [In Chinese]. *Seek Medical and Ask The Medicine*. *10*(11): 281 <http://www.cnki.com.cn/Article/CJFDTotal-QYWA201211302.htm>.

10.Wu LY, Xu X, Ma HM. (2012) Investigation and analysis of influencing factors of irritable bowel syndrome among internal medical students in Wuhan City [In Chinese]. *CHINA MEDICAL HERALD*. *9*(33): 125-27 <https://doi.org/10.3969/j.issn.1673-7210.2012.33.048>.

11.Dong YY, Chen FX, Yu YB, et al. (2013) A School-Based Study with Rome III Criteria on the Prevalencof Functional Gastrointestinal Disorders in Chinese College and University Students. *PloS one*. *8*(1): 125-27 <https://doi.org/10.1371/journal.pone.0054183>.

12.Li M, Lu B, Chu L, Zhou H, Chen MY. (2014) Prevalence and characteristics of dyspepsia among college students in Zhejiang province. *World J Gastroenterol* *20*(13): 3649-54 [https://dx.doi.org/10.3748/w](https://dx.doi.org/10.3748/wjg.v20.i13.3649)

jg.v20.i13.3649.

13.Liu Y, Liu L, Yang Y, et al. (2014) A school-based study of irritable bowel syndrome in medical students in Beijing, China: Prevalence and some related factors. *Gastroenterol Res Pract*. *2014*: 12426

1 <https://doi.org/10.1155/2014/124261>.

14.Yang Y, Liu L, He YX, Zhao HC, Yao SK, Zhang YL. (2015) Gender differences in the prevalence of irritable bowel syndrome and related factors in medical students [In Chinese]. *Journal of China-Japan Friendship Hospital*. *29*(03): 177-79 <https://doi.org/10.3969/j.issn.1001-0025.2015.03.015>.

15.Zhang J, Zhang GL, Liu H, Liu F, Chen WG, Zheng Y. (2015) Morbidity of IBS and its related factors in different major postgraduates [In Chinese] *Chin J Gastroenterol Hepatol.* 24(03): 291

295 <https://kns.cnki.net/kcms/detail/detail.aspx?dbcode=CJFD&dbname=CJFDLAST2015&filename=WCBX20153>017&uniplatform=NZKPT&v=tTCR0K2CdggoSfc_sLdsS3o_R_4I9bW30hhCBoQ9RaHPhpC2UF37Xub6muVmwDMe.

16.Li HL, Liu L, Ren W, et al. (2016) A school-based study of irritable bowel syndrome in college students in Beijing: prevalence and some relative factors [In Chinese]. *Chin J Gastroenterol Hepatol*. *25*(04): 448-51<https://kns.cnki.net/kcms/detail/detail.aspx?dbcode=CJFD&dbname=CJFDLAST2016&filename=WCBX20160402&>uniplatform=NZKPT&v=4gRYzGULNtditd9r_QRe0NY0yrl-fbPEM0XhBC6QjJhj9XX-YGr92G94G_wu6ahp.

17.Wang Y, Jin F, Chi B, et al. (2016) Gender differences in irritable bowel syndrome among medical students at Inner Mongolia Medical University, China: a cross-sectional study. *Psychology, health & medicine*. *21*(8) 964-74 <https://doi.org/10.1080/13548506.2016.1144890>.

18.Yang KD, Jin Y, Yang TF. (2016) Investigation and syndrome differentiation of Irritable bowel syndrome among students in a university in Guangzhou [In Chinese]. *JOURNAL OF NEW CHINESE MEDICINE*. *48*(08): 74-75 <https://doi.org/10.13457/j.cnki.jncm.2016.08.032>.

19.Chen LL, Shen F, Hong LX, et al. (2018) The association of sleep quality with prevalence of irritable bowel syndrome in female college students from the faculty of nursing [In Chinese] *Nursing of Integrated Traditional Chinese and Western Medicine*. *4*(05): 23-26 [https://kns.cnki.net/kcms/detail/detail.aspx?dbcode=CJFD&d](https://kns.cnki.net/kcms/detail/detail.aspx?dbcode=CJFD&dbname=CJFDLAST2016&filename=WCBX20160402&)bname=CJFDLAST2018&filename=ZXHL201805007&uniplatform=NZKPT&v=aeRTx0M5aKs8qH19WwaWRUkgsD083mp2tYn0S03onViFyVU6AivtimZH-aErzZPL.

20.Liu HY, Jiang LP, Wei XX, et al. (2019) Investigation analysis of the prevalence situation and risk factors for irritable bowel syndrome of students in a higher vocational college in Guangxi based on the Rome IV standard [In Chinese]. *Internal Medicine*. *14*(05): 516-18 <https://doi.org/10.16121/j.cnki.cn45-1347/r.2019.05.02>.

21.Chen HH, Hung CH, Kao AW, Hsieh HF. (2021) Exploring Quality of Life, Stress, and Risk Factors Associated with Irritable Bowel Syndrome for Female University Students in Taiwan. *International journal of environmental research and public health*. *18*(8): 3888 <https://doi.org/10.3390/ijerph18083888>.

22.Zhang XJ, Ma SJ, Li R. (2021) Prevalence of irritable bowel syndrome and analysis of related factors in Soochow University [In Chinese]. *Contemporary medical*. *27*(8): 120-23 [https://doi.org/10.3969/j.issn.1009-4393.](https://doi.org/10.3969/j.issn.1009-4393.2021.08.048) 2021.08.048.

# Supplementary Figures and Tables

## Supplementary Figures


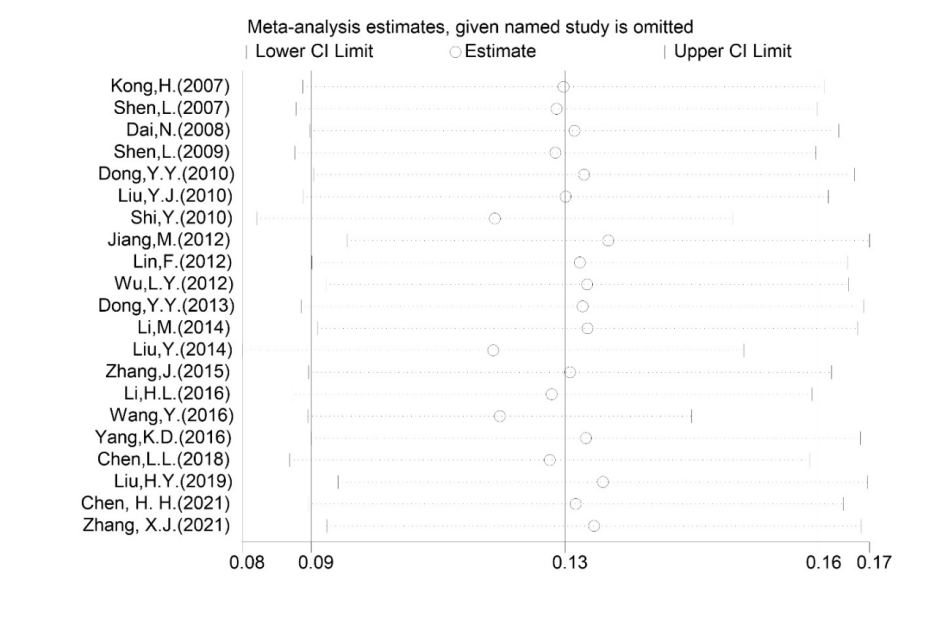


**Supplementary Figure 1.** Sensitivity analysis of eligible studies in the meta-analysis

## Supplementary Tables

**Supplementary Table 1.** Search strategies

| Database | Strategy |
| --- | --- |
| PubMed  (1950-5/31/2021) | (((Irritable OR spastic OR Mucous) AND (bowel OR colon OR colonic OR gastrointestinal)) OR IBS)  AND  (China OR Chinese OR mainland China OR Hong Kong OR Macau OR Macao OR Taiwan)  AND  (college OR University OR undergrad*) |
| MEDLINE (EBSCOhost)  (1950-5/31/2021) | (((Irritable OR spastic OR Mucous) AND (bowel OR colon OR colonic OR gastrointestinal)) OR IBS)  AND  (China OR Chinese OR mainland China OR Hong Kong OR Macau OR Macao OR Taiwan)  AND  (college OR University OR undergrad*) |
| Embase  (1970-5/31/2021) | (((Irritable OR spastic OR Mucous) AND (bowel OR colon OR colonic OR gastrointestinal)) OR IBS)  AND  (China OR Chinese OR mainland China OR Hong Kong OR Macau OR Macao OR Taiwan)  AND  (college OR University OR undergrad*) |
| CINAHL Complete  (1937-5/31/2021) | (((Irritable OR spastic OR Mucous) AND (bowel OR colon OR colonic OR gastrointestinal)) OR IBS)  AND  (China OR Chinese OR mainland China OR Hong Kong OR Macau OR Macao OR Taiwan)  AND  (college OR University OR undergrad*) |
| CNKI  (中国知网)  (1999-5/31/2021) | FT=(“肠易激综合征”+“肠道易激综合症”+“肠易激惹综合征”+“结肠易激综合征”+“肠易激综合症”+“肠道易激综合征”+“肠应激综合征”)  AND  FT=(“大学生”+“高校”+“高等院校”+“本科生”) |
| Weipu via VIP (维普)  (1989-5/31/2021) | U= (肠易激综合征 OR 肠道易激综合症 OR 肠易激惹综合征 OR 结肠易激综合征 OR 肠易激综合症 OR 肠道易激综合征 OR 肠应激综合征)  AND  U= (大学生 OR 高校 OR 高等院校 OR 本科生) |
| WANFANG  (万方数据)  (1989-5/31/2021) | 全部: (肠易激综合征OR肠道易激综合症OR肠易激惹综合征OR结肠易激综合征OR肠易激综合症OR肠道易激综合征OR肠应激综合征)  AND  (大学生OR高校OR高等院校OR本科生) |

**Supplementary Table 2.** The quality assessment of included studies based on the Joanna Briggs Institute’s checklist (n=22)

| **Study** | **Q1^a^** | **Q2^b^** | **Q3^c^** | **Q4^d^** | **Q5^e^** | **Q6^f^** | **Q7^g^** | **Q8^h^** | **Q9^i^** | **Score** |
| --- | --- | --- | --- | --- | --- | --- | --- | --- | --- | --- |
| Kong, H (2007) | Y^j^ | Y | Y | N^k^ | Y | Y | Y | Y | Y | 8 |
| Shen, L (2007) | Y | Y | Y | Y | Y | Y | Y | Y | Y | 9 |
| Dai, N (2008) | Y | Y | Y | Y | Y | Y | Y | Y | Y | 9 |
| Shen, L. (2009) | Y | Y | Y | Y | Y | Y | Y | Y | Y | 9 |
| Liu, Y.J. (2010) | Y | Y | Y | N | Y | Y | Y | Y | Y | 8 |
| Shi, Y. (2010) | Y | Y | Y | Y | Y | Y | U^l^ | Y | Y | 8 |
| Dong, Y.Y. (2010) | Y | Y | Y | Y | Y | Y | Y | Y | Y | 9 |
| Jiang, M. (2012) | Y | Y | Y | N | Y | Y | U | Y | Y | 7 |
| Lin, F. (2012) | Y | Y | Y | Y | Y | Y | U | Y | Y | 8 |
| Wu, L.Y. (2012) | Y | N | U | Y | Y | Y | U | Y | Y | 6 |
| Dong, Y.Y. (2013) | Y | Y | Y | Y | Y | Y | Y | Y | Y | 9 |
| Li, M. (2014) | Y | U | Y | Y | Y | Y | U | Y | N | 6 |
| Liu, Y. (2014) | Y | Y | Y | Y | Y | Y | U | Y | Y | 8 |
| Yang, Y (2015) | Y | Y | Y | N | Y | Y | U | Y | Y | 7 |
| Zhang, J. (2015) | Y | Y | Y | Y | Y | Y | Y | Y | Y | 9 |
| Li, H.L. (2016) | Y | Y | Y | Y | Y | Y | U | Y | Y | 8 |
| Wang, Y. (2016) | Y | U | Y | Y | Y | Y | Y | Y | Y | 8 |
| Yang, K.D. (2016) | Y | Y | Y | N | Y | Y | U | Y | Y | 7 |
| Chen, L.L. (2018) | Y | Y | Y | N | Y | Y | U | Y | Y | 7 |
| Liu, H.Y. (2019) | Y | Y | Y | N | Y | Y | Y | Y | Y | 8 |
| Chen, H.H. (2021) | Y | N | Y | Y | Y | Y | U | Y | Y | 7 |
| Zhang, X.J. (2021) | Y | Y | Y | Y | Y | Y | U | Y | Y | 8 |

*Note*:

^a^Question 1: Was the sample frame appropriate to address the target population?

^b^Question 2: Were study participants recruited in an appropriate way?

^c^Question 3: Was the sample size adequate?

^d^Question 4: Were the study subjects and setting described in detail?

^e^Question 5: Was data analysis conducted with sufficient coverage of the identified sample?

^f^Question 6: Were valid methods used for the identification of the condition?

^g^Question 7: Was the condition measured in a standard, reliable way for all participants?

^h^Question 8: Was there appropriate statistical analysis?

^i^Question 9: Was the response rate adequate, and if not, was the low response rate managed appropriately?

^j^Y: Yes

^k^N: No

^l^U: Unclear

**Supplementary Table 3 (A).** Subgroup analyses of the pooled prevalence according to Rome Ⅱ

| **Subgroup analysis** | **Diagnostic**  **Criteria** | **Studies(n)** | **Sample Size** | **Pooled**  **Prevalence (%)** | **95% CI** | | **I2(%) within subgroup** | ***p-*value across subgroups** |
| --- | --- | --- | --- | --- | --- | --- | --- | --- |
|  |  |  |  |  | **Lower** | **Upper** |  |  |
| Education level | Rome Ⅱ |  |  |  |  |  |  | **<0.001** |
| undergraduate |  | 4 | 3,898 | 9.95 | 5.87 | 16.36 | 95.18 |  |
| postgraduate |  | 2 | 484 | 13.32 | 9.15 | 19.01 | 52.93 |  |
| Gender | Rome Ⅱ |  |  |  |  |  |  | **<0.001** |
| Female |  | 5 | 2,386 | 13.76 | 10.31 | 18.12 | 83.48 |  |
| Male |  | 5 | 1,996 | 10.17 | 7.17 | 14.22 | 82.05 |  |
| Major | Rome Ⅱ |  |  |  |  |  |  | **<0.001** |
| Medicine |  | 4 | 1,525 | 11.48 | 9.36 | 14.00 | 46.34 |  |
| Non-medicine |  | 3 | 1,754 | 14.10 | 6.43 | 28.17 | 95.79 |  |
| Mixed |  | 1 | 1,121 | 4.82 | 3.71 | 6.24 | 0.00 |  |
| Region | Rome Ⅱ |  |  |  |  |  |  | **<0.001** |
| Central China |  | 2 | 822 | 15.57 | 13.25 | 18.21 | 0.00 |  |
| East China |  | 3 | 3,560 | 7.99 | 4.90 | 12.76 | 92.55 |  |
| Survey year | Rome Ⅱ |  |  |  |  |  |  | **<0.001** |
| 2005-2010 |  | 2 | 2,425 | 23.00 | 10.69 | 42.71 | 97.96 |  |
| 2010-2021 |  | 2 | 3,333 | 6.95 | 1.07 | 34.11 | 99.37 |  |

*Note*: Boldface indicates statistical significance (*p*<0.05).

CI, confidence interval.

**Supplementary Table 3 (B).** Subgroup analyses of the pooled prevalence according to Rome Ⅲ

| **Subgroup analysis** | **Diagnostic**  **Criteria** | **Studies(n)** | **Sample Size** | **Pooled**  **Prevalence (%)** | **95% CI** | | **I2(%) within subgroup** | ***p-*value across subgroups** |
| --- | --- | --- | --- | --- | --- | --- | --- | --- |
|  |  |  |  |  | **Lower** | **Upper** |  |  |
| Education level | Rome Ⅲ |  |  |  |  |  |  | **<0.001** |
| Junior college |  | 3 | 2,437 | 11.61 | 6.99 | 18.68 | 94.67 |  |
| Undergraduate |  | 9 | 18,324 | 13.16 | 8.10 | 20.68 | 99.40 |  |
| Postgraduate |  | 1 | 441 | 11.56 | 8.90 | 14.90 | 0.00 |  |
| Gender | Rome Ⅲ |  |  |  |  |  |  | **<0.001** |
| Female |  | 10 | 13,763 | 16.08 | 10.94 | 23.02 | 98.81 |  |
| Male |  | 8 | 6,070 | 12.81 | 7.12 | 21.95 | 98.47 |  |
| Major | Rome Ⅲ |  |  |  |  |  |  | **<0.001** |
| Medicine |  | 10 | 8,191 | 13.44 | 8.40 | 20.82 | 0.70 |  |
| Non-medicine |  | 6 | 7,043 | 11.49 | 8.76 | 14.93 | 0.13 |  |
| Mixed |  | 4 | 9,435 | 8.24 | 2.93 | 21.08 | 1.18 |  |
| Region | Rome Ⅲ |  |  |  |  |  |  | **<0.001** |
| Central China |  | 2 | 2,157 | 15.83 | 2.89 | 54.29 | 97.91 |  |
| East China |  | 6 | 9,057 | 10.58 | 8.44 | 13.19 | 92.77 |  |
| North China |  | 3 | 3,232 | 17.66 | 7.37 | 36.64 | 99.03 |  |
| Northwest China |  | 2 | 6,546 | 19.10 | 7.02 | 42.46 | 98.30 |  |
| South China |  | 2 | 3,186 | 3.19 | 0.51 | 17.38 | 92.83 |  |
| Survey year | Rome Ⅲ |  |  |  |  |  |  | **<0.001** |
| 2005-2010 |  | 5 | 4,851 | 11.67 | 9.12 | 14.81 | 87.64 |  |
| 2010-2021 |  | 9 | 15,822 | 11.93 | 6.81 | 20.05 | 99.29 |  |
| Anxiety | Rome Ⅲ |  |  |  |  |  |  | **<0.001** |
| No |  | 4 | 9,236 | 10.70 | 3.90 | 26.12 | 99.41 |  |
| Yes |  | 4 | 1,353 | 23.55 | 10.64 | 44.36 | 97.42 |  |
| Depression | Rome Ⅲ |  |  |  |  |  |  | **<0.001** |
| No |  | 3 | 8,950 | 12.89 | 4.09 | 33.93 | 99.57 |  |
| Yes |  | 3 | 1,482 | 26.21 | 9.85 | 53.60 | 98.31 |  |
| Drinking | Rome Ⅲ |  |  |  |  |  |  | **<0.001** |
| No |  | 2 | 918 | 19.20 | 4.87 | 52.45 | 97.38 |  |
| Yes |  | 2 | 289 | 17.29 | 9.08 | 30.45 | 73.11 |  |

*Note*: Boldface indicates statistical significance (*p*<0.05).

CI, confidence interval.

**Supplementary Table 3 (C).** Subgroup analyses of the pooled prevalence according to Rome Ⅳ

| **Subgroup analysis** | **Diagnostic**  **Criteria** | **Studies(n)** | **Sample Size** | **Pooled**  **Prevalence (%)** | **95% CI** | | **I2(%) within subgroup** | ***p-*value across subgroups** |
| --- | --- | --- | --- | --- | --- | --- | --- | --- |
|  |  |  |  |  | **Lower** | **Upper** |  |  |
| Gender | Rome Ⅳ |  |  |  |  |  |  | **<0.001** |
| Female |  | 2 | 2,707 | 3.78 | 2.33 | 6.08 | 81.21 |  |
| Male |  | 2 | 1,126 | 3.02 | 1.01 | 8.63 | 89.07 |  |

*Note*: Boldface indicates statistical significance (p<0.05).

CI, confidence interval.

**Supplementary Table 4.** Pooled odds ratio for IBS in Chinese university students

| **Variable** | **Studies(n)** | **Sample Size(n)** | **Pooled Odds Ratio (95%CI)** | ***P*-value** |
| --- | --- | --- | --- | --- |
| Sleep disorder  (yes vs.no) | 4 | 2,883 | **1.48 (1.02,2.15)** | **0.04** |
| Anxiety  (yes vs.no) | 6 | 13,698 | **2.35 (2.03,2.72)** | **<0.001** |
| Depression  (yes vs.no) | 5 | 13,541 | **2.15 (1.88,2.47)** | **<0.001** |
| Drinking  (yes vs.no) | 4 | 4,541 | 1.13 (0.81,1.57) | 0.47 |
| Exercise  (yes vs.no) | 4 | 9,221 | 0.78 (0.56,1.09) | 0.15 |
| Gender  (female vs. male) | 14 | 24,565 | **1.36 (1.08,1.69)** | **0.008** |
| Major  (medicine vs. non-medicine) | 9 | 12,795 | 0.78 (0.59,1.03) | 0.08 |
| Smoking  (yes vs.no) | 4 | 3,122 | 1.21 (0.45,3.26) | 0.70 |

*Note*: Boldface indicates statistical significance (*p*<0.05).

CI, confidence interval.
